# Zebrafish sperm outsource activation to eggs’ protease-activated receptors

**DOI:** 10.1371/journal.pbio.3003214

**Published:** 2025-06-18

**Authors:** Rafael A. Fissore, Emily M. Lopes, Francesca Carpentiero

**Affiliations:** 1 Department of Veterinary and Animal Sciences, University of Massachusetts, Amherst, Massachusetts, United States of America; 2 Molecular and Cellular Biology Graduate Program, University of Massachusetts, Amherst, Massachusetts, United States of America

## Abstract

The calcium surge that starts embryogenesis varies across species. A new study in PLOS Biology presents a novel mechanism of egg activation in zebrafish, wherein protease-activated receptors mediate a calcium signal that initiates egg activation before sperm entry.

## Teaser

*The calcium surge that starts embryogenesis varies across species and is elusive in many. A new study in* PLOS Biology *shows that zebrafish eggs self-activate, initiating a protease-activated receptor calcium wave and uncovering a novel pathway in egg activation.*

**Fig 1:**
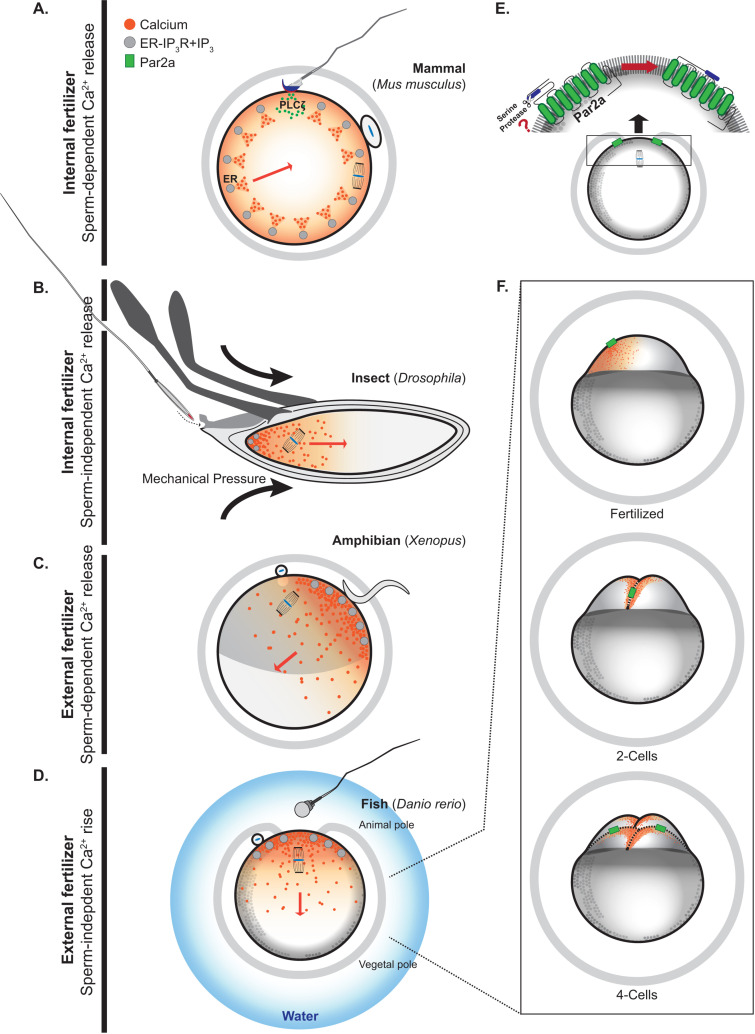
A Par-induced Ca^2+^ rise triggers zebrafish egg activation independently of sperm entry. A rise in intracellular Ca^2+^ is a universal trigger of egg activation across animal species, occurring in internal and external fertilizers, either coinciding with or independent of sperm entry. These Ca^2+^ increases typically originate from the endoplasmic reticulum stores via inositol 1,4,5-trisphosphate (IP_3_) production and IP_3_R-mediated Ca^2+^ release. Although external Ca^2+^ is left out of the figure, it contributes directly or indirectly to the activation-associated Ca^2+^ signals across taxa. **(A)** *Mouse*: An internal fertilizer where sperm entry initiates sustained Ca^2+^ oscillations via sperm-derived PLCζ. **(B)** *Drosophila*: An internal fertilizer, but egg activation triggered by mechanical stimuli following ovulation precedes fertilization. **(C)** *Amphibians:* External fertilizing species where sperm interaction with an unidentified oolemma receptor triggers the Ca^2+^ wave. **(D)** *Zebrafish*: An external fertilizing species where Ca^2+^ release and egg activation occur upon spawning and following contact with water and activation of a protease-activated receptor 2a (Par-2a). **(E)** Speculative localization of Par-2a and associated G proteins in the zebrafish egg cortex before fertilization; the activating serine proteases and precise ligand changes remain unidentified. **(F)** Post-activation Ca^2+^ dynamics in zebrafish embryos: each cleavage division is driven by a localized Ca^2+^ rise at the cleavage furrow. The extent to which Par-2a and IP_3_ signaling contribute to these post-activation Ca^2+^ transients will require additional confirmation. Shown: 1-cell zygote, 2-cell, and 4-cell stages.

Fertilization marks the initiation of development, as gametes from opposite sexes unite to produce progeny and ensure the continuity of the species. An early and remarkable event associated with this union is egg activation, the first and transitional step in development that transforms two haploid cells into a single, developmentally competent zygote. The necessary remodeling, unfolding in minutes or hours, is coordinated by a specialized calcium (Ca^2+^) signal within the egg [1,2]: a unifying feature of egg activation across a range of fertilization strategies (Fig 1). In a new article in this issue, Ma and Carney [3] uncover how zebrafish (*Danio rerio*) eggs autonomously initiate a protease-activated receptor 2 (Par-2)-triggered Ca^2+^  elevation that spreads as a wave and reaches the antipode within 1 minute and represents the activating signal. They also investigate the Par-2 signaling pathway and its association with the Ca^2+^ transients of cleavage divisions.

Animal species can be broadly categorized as internal or external fertilizers. Internal fertilization occurs in the female reproductive organs and is typically observed in but not limited to mammals, birds, reptiles, insects, and nematodes. In mammals and nematodes, sperm entry and egg activation are tightly coupled, with sperm entry directly triggering the activating Ca^2+^ signal [1]. Insects, however, often exhibit a temporal separation between fertilization and activation. In *Drosophila*, egg activation is completed before fertilization, whereas in other insects, sperm entry precedes activation, yet in others, it does not occur at all, leading to parthenogenesis.

Bony fish, including zebrafish, are external fertilizers, implying sperm entry and activation occur outside the body, typically in water. Other external fertilizers include amphibians, echinoderms, and other marine invertebrates [1]. To ensure fertilization, external fertilizers rely on strategies such as amplexus, jelly coat, sperm chemotaxis, and rapid signaling at the egg plasma membrane to accomplish interspecific sperm recognition and block polyspermy, respectively. In these species, including the medaka fish, *Oryzias latipes*, contact with or entry of sperm triggers egg activation. However, the egg activation mechanism in zebrafish has remained unclear despite the evidence that sperm entry and activation are separate events [4], partly because the activation onset is immediate after spawning and closely followed by the release of the male’s milt, obscuring the sperm’s role in this process. In their new study [3], Ma and Carney advance compelling evidence that zebrafish eggs activate independently from the sperm via a Par-2 pathway. Their results include preventing egg activation events after natural spawning with or without sperm by serine protease inhibitors and the failure of CRISPR/Cas9-generated *par2a* homozygous mutant female eggs to activate under similar circumstances. These findings point to a novel, protease-activated receptor-dependent mechanism of egg activation in zebrafish. Research in other species has provided convincing evidence for the participation of proteases and their receptors in egg activation. However, the underlying pathways remained undiscerned, or their essential role was masked by redundancy [5].

Ma and Carney [3] also explored the Par-2 signaling pathway during zebrafish fertilization. Pars are G protein-coupled receptors, and their activation usually follows partial receptor cleavage by a proteinase that unleashes a “tethered ligand”, or another activation mechanism [6]. This leads to downstream signaling, inositol 1,4,5-trisphosphate (IP_3_) production, and Ca^2+^ release via the IP_3_ receptor (IP_3_R) [5,6]. The role of IP_3_ signaling in egg activation is conserved across species, including zebrafish, where IP_3_R function has been demonstrated using broad antagonists [7]. In this study [3], eggs treated with 2-aminoethyl diphenylborinate or a molecular IP_3_R inhibitor were prevented from becoming activated after spawning. Further, IP_3_ injection rescued activation in protease inhibitor-treated or *par-2a* mutant eggs, demonstrating that Par signaling promotes egg activation through the canonical IP₃-Ca^2+^ pathway.

The involvement of IP_3_ signaling suggests a role for phosphoinositide-hydrolyzing enzymes such as phospholipases (PLCs), which are well characterized in fertilization. In marine invertebrates and *Xenopus*, IP_3_ production is controlled by PLC and Src kinases, but the upstream receptor(s) remain unknown [5,8,9]. In mammals, a sperm-specific PLC is released into the cytosol upon gamete fusion, triggering Ca^2+^ oscillations [10]. In zebrafish, the new transcriptomic studies in eggs identified robust expression of Gαq family members (*gna11a* and *gna11b*) along with PLCβ isozymes (*plcb3* and *plcb4*), consistent with canonical Par signaling in other systems [3]. Pharmacological inhibition of Gαq/11 and PLCβ activity yielded variable outcomes, and even though the PLCβ inhibitor U-73122 blocked egg activation, the precise molecular players downstream of Par-2 need to be defined with genetic and molecular approaches.

Following the initial Ca^2+^ surge, activated zebrafish eggs undergo meroblastic cleavages, each accompanied by a discrete Ca^2+^ transient. Similar IP₃R-dependent Ca^2+^ spikes occur in early cleavage in *Xenopus* [11]. Ma and Carney argue that Par-2 signaling is necessary for the blastomere cleavages because pharmacological inhibition of Par-2 impairs them and cannot be rescued by exogenous IP₃. However, IP₃ injection rescued activation and, in many cases, near-normal blastomere division, when *par2a* is genetically inactivated, as is the case with *par2a* mutant eggs. Therefore, despite the additional demonstration that Par-2 localizes to the cleavage furrows, the extent of Par-2 and IP_3_ signaling contribution to blastomere division requires clarification.

Overall, Ma and Carney [3] have demonstrated that zebrafish egg activation is independent of sperm entry and initiated by a Par-2-mediated, IP_3_-induced Ca^2+^ release. This signaling triggers egg activation, but the role in blastomere division remains inconclusive. Open questions include the identity of the serine protease that activates the tethered Par-2 ligand(s) and the localization of Par-2 in the egg membrane. Importantly, this study identifies a plasma membrane receptor involved in activating embryo development and confirms the essential role of the IP_3_-IP_3_R-Ca^2+^ axis in this process. The conservation of Par-2-like signaling should be extended, especially in species of external fertilizers where tantalizing evidence is already available. The findings here and the supporting literature remind us of nature’s inexhaustible ingenuity to devise diverse yet convergent solutions for initiating life across species and environments, even if we are unaware of many aspects of their origin and regulation.

## Abbreviations

IP_3_: inositol 1,4,5-trisphosphate
IP_3_R: IP_3_ receptor
Par-2: protease-activated receptor 2
Par-2a: protease-activated receptor 2a
PLCs: phospholipases
